# Generation of α-1,3-galactosyltransferase knocked-out transgenic cloned pigs with knocked-in five human genes

**DOI:** 10.1007/s11248-016-9979-8

**Published:** 2016-08-23

**Authors:** Dae-Jin Kwon, Dong-Hwan Kim, In-Sul Hwang, Dong-Ern Kim, Hyung-Joo Kim, Jang-Seong Kim, Kichoon Lee, Gi-Sun Im, Jeong-Woong Lee, Seongsoo Hwang

**Affiliations:** 1 National Institute of Animal Science, Rural Development Administration, Wanju, Jeollabuk-do 55365 Republic of Korea; 2 Biotherapeutics Translational Research Center, Korea Research Institute of Bioscience and Biotechnology, Daejeon, 34141 Republic of Korea; 3 Department of Functional Genomics, University of Science and Technology, Daejeon, 34113 Republic of Korea; 4 Department of Animal Sciences, The Ohio State University, Columbus, OH 43210 USA

**Keywords:** Multi-transgenic pigs, Xenotransplantation, Immune rejection, Leucopenia, Thrombocytopenia

## Abstract

Recent progress in genetic manipulation of pigs designated for xenotransplantation ha6s shown considerable promise on xenograft survival in primates. However, genetic modification of multiple genes in donor pigs by knock-out and knock-in technologies, aiming to enhance immunological tolerance against transplanted organs in the recipients, has not been evaluated for health issues of donor pigs. We produced transgenic Massachusetts General Hospital piglets by knocking-out the α-1,3-galactosyltransferase (GT) gene and by simultaneously knocking-in an expression cassette containing five different human genes including, DAF, CD39, TFPI, C1 inhibitor (C1-INH), and TNFAIP3 (A20) [GT^−(DAF/CD39/TFPI/C1-INH/TNFAIP3)/+^] that are connected by 2A peptide cleavage sequences to release individual proteins from a single translational product. All five individual protein products were successfully produced as determined by western blotting of umbilical cords from the newborn transgenic pigs. Although gross observation and histological examination revealed no significant pathological abnormality in transgenic piglets, hematological examination found that the transgenic piglets had abnormally low numbers of platelets and WBCs, including neutrophils, eosinophils, basophils, and lymphocytes. However, transgenic piglets had similar numbers of RBC and values of parameters related to RBC compared to the control littermate piglets. These data suggest that transgenic expression of those human genes in pigs impaired hematopoiesis except for erythropoiesis. In conclusion, our data suggest that transgenic expression of up to five different genes can be efficiently achieved and provide the basis for determining optimal dosages of transgene expression and combinations of the transgenes to warrant production of transgenic donor pigs without health issues.

**Electronic supplementary material:**

The online version of this article (doi:10.1007/s11248-016-9979-8) contains supplementary material, which is available to authorized users.

## Introduction

Xenotransplantation of organs from a readily available animal species, such as the pig, into humans has been considered as a potential solution to the critical shortage of organs from deceased human donors for transplantation (Yamada et al. 2005; Griesemer et al. 2009). However, the immune rejection response due to species disparity between pigs and primates is the major hurdle in successful xenograft transplantation. A great amount of effort has been applied to prolong survival periods of transplanted xenografts, including the deletion of galactose-α-1,3-galactose antigens (α-Gal) by knockout of the α-1,3-galactosyltransferase (GT KO) gene (Lai 2002) and simultaneous inhibition of human complement activation by the introduction of human complement-regulatory genes, such as CD46, CD55 (decay accelerating factor; DAF) (Lee et al. 2011), and CD59, in the pigs used for xenotransplantation (Lambrigts et al. 1998; McGregor et al. 2004). The classical pathway complement-regulatory protein C1 inhibitor (C1-INH) has also been reported to be able to prevent complement-mediated activation of xenogeneic endothelial cells (Dalmasso and Platt 1993; Wheeler et al. 2012). Due to the presence of non-Gal antibodies and molecular incompatibilities between coagulation systems of primates and pigs, thrombotic microangiopathy has often developed in the vascularized graft tissues. Moreover, inflammatory responses against pig organs may evoke some acute vascular rejection. However, these problems may be overcome by the introduction of human coagulation-regulatory genes, such as thrombomodulin and tissue factor pathway inhibitor (TFPI) (Lee et al. 2011), or human anti-inflammatory genes, such as hemeoxygenase-1 and CD39 (Wheeler et al. 2012) and/or tumor necrosis factor-α induced protein 3 (TNFAIP3; A20) (Daniel et al. 2004; Oropeza et al. 2009), respectively. As a result, significant progress has been made in that cardiac xenograft from GT KO pigs with human complement regulatory protein (hCD46, membrane cofactor protein) and human thrombomodulin molecules to baboons had survived for over 900 days (Mohiuddin et al. 2015). Also, an efficacy of renal xenotransplantation has been established with survival and organ functionality extending over 227 days with GT KO/CD55 transgenic pig-to-macaque (Higginbotham et al. 2015).

Although the expression of these human genes in the pigs for xeno-organ sources has revealed some beneficial effects in the success of xenotransplantation, it is not clear and remains to be determined what combination of genes would be better to develop a state of immunological tolerance against transplanted organs in the recipients. To achieve this goal, genetic introduction of multiple genes into pigs would be required. Thus, there has been an urgent need for the development of an efficient strategy for the introduction of multiple human genes in those pigs.

In the present study, we attempted to produce a transgenic piglet with GT KO and simultaneously expressing multiple transgenes, including human DAF, hCD39, hTFPI, hC1-INH, and hTNFAIP3. Here, we report technical advantages of this approach to generate the MGH GT^−(DAF/CD39/TFPI/C1-INH/TNFAIP3)/+^ piglets as well as some health issues of these transgenic pigs.

## Materials and methods

### General information

All chemicals were purchased from Sigma-Aldrich Corp. (St. Louis, MO, USA) and all restriction enzymes were from New England Biolabs (Ipswich, MA, USA) unless otherwise stated. The study protocol and standard operating procedures for the treatments of the pigs used in the present study were reviewed and approved by the Institutional Animal Care and Use Committee of the National Institute of Animal Science, Rural Development Administration of Korea (approval number: 2012-D-001, NIAS2015-671).

### Construction of a zinc finger nuclease (ZFN) vector

A pair of ZFN vectors was constructed for targeting exon 9 of the GT gene. The coding sequence of DNA binding domain, used in the previous study (Hauschild et al. 2011), was synthesized (Bioneer Inc., Daejeon, Korea) and inserted into a ZFN expression vector, pST1374, in the *Bam*HI and *Xba*I sites to make a PaGE9L/R vector. Cloned DNA sequences were confirmed by DNA sequencing analysis (Genotech, Daejeon, Korea).

### Polymerase chain reaction and gene cloning

Total RNA was isolated from human umbilical vein endothelial cells using TRIZOL reagent (Life Technologies, Carlsbad, CA, USA) and used for reverse transcription by SuperScript III first-strand synthesis system (Invitrogen, Life Technologies, Carlsbad, CA, USA) according to the manufacturer’s instruction. Complementary DNAs coding for human DAF, CD39, TFPI, C1-INH, or TNFAIP3 were amplified by PCR with *pfu* DNA polymerase (Enzynomics, Daejeon, Korea) using specific primer sets (Suppl. Table 1) and human cDNAs as the template. PCR conditions were as follows: hDAF, initial denaturation at 95 °C for 5 min; 35 cycles of 94 °C for 30 s, 65 °C for 30 s, and 72 °C for 2 min; followed by a final extension at 72 °C for 5 min; hCD39, initial denaturation at 95 °C for 5 min; 35 cycles of 94 °C for 30 s, 64 °C for 30 s, and 72 °C for 2 min; followed by a final extension at 72 °C for 5 min; hTFPI, initial denaturation at 95 °C for 5 min; 35 cycles of 94 °C for 30 s, 62 °C for 30 s, and 72 °C for 1 min; followed by a final extension at 72 °C for 5 min; hC1-INH, initial denaturation at 95 °C for 5 min; 35 cycles of 94 °C for 30 s, 64 °C for 30 s, and 72 °C for 2 min; followed by a final extension at 72 °C for 5 min; hTNFAIP3, initial denaturation at 95 °C for 5 min; 35 cycles of 94 °C for 30 s, 64 °C for 30 s, and 72 °C for 3 min; followed by a final extension at 72 °C for 5 min. Amplified DNA fragments were cloned into pGEM-easy TA cloning vector (Promega, Madison, WI, USA), and all cloned DNA fragments were confirmed by DNA sequencing analysis (Genotech).

### Construction of the pBS-2A5 vector

Multiple ‘self cleaving’ 2A peptide sequences (Higginbotham et al. 2015), including P2A, T2A, E2A, or F2A, were synthesized (Bioneer Inc.) and then inserted into the *Sac*II and *Xho*I restriction enzyme sites of the pBluescript II KS (Addgene, Cambridge, MA, USA) plasmid to make a pBS-2A vector. Porcine intercellular adhesion molecule 2 (ICAM2) promoter sequences were amplified by PCR with *pfu* DNA polymerase (Enzynomics) and a specific primer set (Suppl. Table 1) using porcine genomic DNA as the template. PCR was performed under the following cycles: initial denaturation at 95 °C for 5 min; 35 cycles of 94 °C for 30 s, 66 °C for 30 s, and 72 °C for 1 min; followed by a final extension at 72 °C for 5 min. The PCR product was inserted into pGEM-easy TA cloning vector to make pTA-ICAM2. The pTA-ICAM2 vector was then cut by *Xho*I and *Sal*I, and the digested DNA fragment was subcloned into the corresponding sites of the pBS-2A vector. Next, five human genes were digested with the following restriction enzymes (DAF, *Eco*RV and *Avr*II; CD39, *Sna*BI and *Spe*I; TFPI, *Sma*I and *Avr*II; C1-INH, *Stu*I and *Nhe*II, and hTNFAIP3, *Nru*I and *Sal*I, respectively). The digested DNA fragments were subcloned into pBS-2A vector to make a pBS-ICAM2-2A5 vector. Construction of the vector was confirmed by digestion patterns with the following restriction enzymes: *Xba*I, *Stu*I, *Nhe*I, *Hind*II, and *Bam*HI. All cloned DNA sequences were confirmed by DNA sequencing analysis (Genotech).

### Construction of the pLNDT-2A5-Gal expression vector

**Fig. 1:**
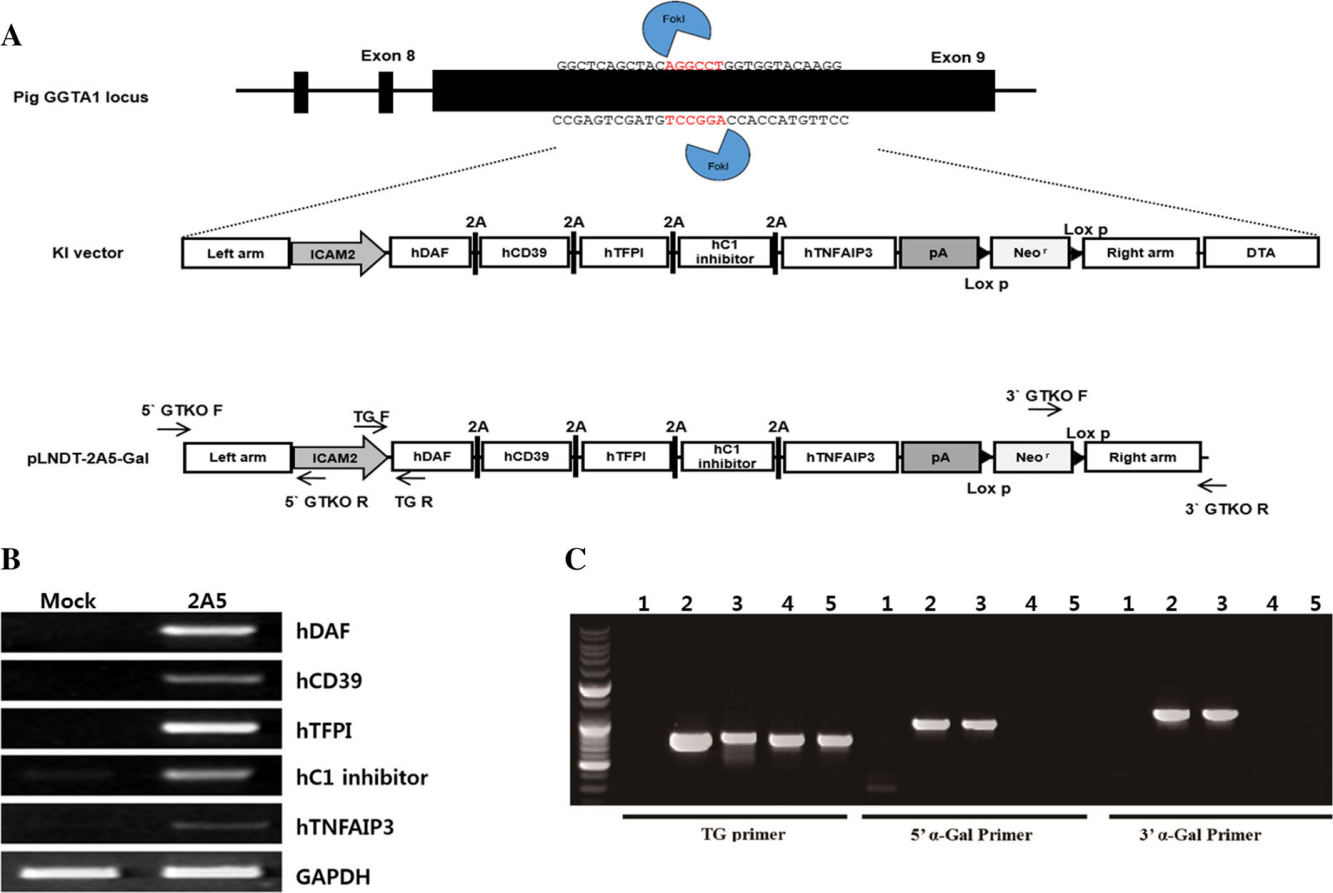
Construction of multiple gene expression vector and identification of GGTA1 (GT) KO/multiple gene KI cell lines. **a** Schematic of a ZFN-based KI process. The KI vector pLNDT-2A5-Gal has an expression unit for multiple genes with 5′ and 3′ α-gal homology arms at its both end. In the *bottom* of this figure, the structure of working pLNDT-2A5Gal vector is shown. Primers used for identification of KI cell clones are shown above and below the constructs. **b** Confirmation of the multiple genes in porcine aortic endothelial cells (PAECs) by RT-PCR. Established the ICAM2-2A5 vector which is the multiple gene expression vector connected by 2A was transfected into PAECs and verified expression of the multiple genes by 2A system before establishment of cell lines. NC: normal PAECs; 2A5: PAECs transfected ICAM2-2A5 vector. **c** Genotyping of selected cell lines. Targeting vector was transfected into ear fibroblasts from MGH minipigs (mpEF). Four cell lines were established and confirmed by 3 of specific primer sets: 0.8 kb of TG primer sets; 1 kb of 5′ a-Gal primer sets; 1.1 kb of 3′ a-gal primer stets. Among the four cell lines, only two of them were confirmed as transgenic cell lines. *1* normal pig genomic DNA; #*2* and *3* transgenic and Knock-in cells; #*4* and *5* transgenic cell lines

Genomic DNA from the Massachusetts General Hospital minipig (MGH minipig) was isolated using a genomic DNA extraction kit (Qiagen, Valencia, CA, USA) according to the manufacturer’s protocol. 1 kb of 5′ α-Gal and 1.1 kb of 3′ α-Gal fragments were amplified by PCR with *pfu* DNA polymerase (Enzynomics) using specific primer sets (Suppl. Table 1) and porcine genomic DNA as the template. The PCR cycles were as follows: initial denaturation at 95 °C for 5 min; 35 cycles of 94 °C for 30 s, 62 °C for 30 s and 72 °C for 1 min; followed by a final extension at 72 °C for 5 min. The PCR products were inserted into a pGEM-easy TA cloning vector to make pTA-5′ α-Gal and pTA-3′ α-Gal, respectively. The pTA-5′ α-Gal and pTA-3′ α-Gal vectors were digested with *Not*I/*Xho*I or *Sal*I/*Asc*I restriction enzymes, respectively, and were subsequently inserted into the predigested pLNDT vector, which contains neomycin as a positive selection marker and diphtheria toxin A as a negative selection marker, to make a α-Gal targeting vector, pLNDT-Gal. Finally, pBS-2A5 vector was digested with *Xho*I and *Pac*I restriction enzymes, and the digested DNA fragment was subcloned into the corresponding restriction sites of pLNDT-Gal to make a pLNDT-2A5-Gal vector (Fig. 1a). All cloned DNA sequences were confirmed by DNA sequencing analysis (Genotech).

### Confirmation of expression of multiple human genes in pig cell lines

Before establishment of cell lines, the constructed ICAM2-2A5 vector was transfected into porcine aortic endothelial cells (PAECs) to verify whether the multiple human genes are expressed well in pig cells. To confirm the expression of each transgene, after the vector transfection, total RNA was isolated using TRIZOL reagent, according to the manufacturer’s protocol, and was used for cDNA synthesis. PCR was performed using a primer set for each transgene (Suppl. Table 1) and cDNA as the template. The PCR cycle was as follows: initial denaturation at 95 °C for 5 min; 22-36 cycles of 94 °C for 30 s, the optimized annealing temperature for 30 s, and 72 °C for 30 s; followed by a final extension at 72 °C for 5 min.

### Construction of knock-in cell line

Ear fibroblasts obtained from the MGH minipigs (mpEF) were cultured in Dulbecco’s modified Eagle’s medium (WelGene, Gyeongsan-si, Korea) containing 20 % fetal bovine serum, 1 mM of non-essential amino acid (GIBCO), 1 mM of sodium pyruvate (WelGene), 55 μM of β-mercaptoethanol (Life Technologies), and 1 % antibiotics (100 U/ml penicillin and 100 μg/ml streptomycin; Life Technologies) at 37 °C in an atmosphere of 5 % CO_2_. mpEF cells (1 × 10^6^ cells/ml) were co-transfected with 4 ug of a pLNDT-2A5-Gal vector, which was linearized by a *Not*I restriction enzyme and purified by phenol:chloroform reagent, and 2 ug of a pair of ZFN vectors using the Amaxa nuclofector system with U-023 program (Lonza, Basel, Switzerland) and seeded into 48-well plates. After 24 h, transfected cells were selected by 300 μg/ml of neomycin reagent (Invitrogen) for 9 days and each of the single colonies were cultured. Genomic DNA was extracted from the candidate mpEF transfectants using a DNeasy extraction kit (Qiagen). PCR was performed using primer sets for transgenes (TG), 5′ α-Gal and/or 3′ α-Gal (Suppl. Table 2) and purified genomic DNAs as the template. PCR cycles were as follows: initial denaturation at 95 °C for 5 min; 30 cycles of 94 °C for 30 s, 55 °C (for TG), 65 °C (for 5′ α-Gal) or 50 °C (for 3′ α-Gal) for 30 s, and 72 °C for 1 min; followed by a final extension at 72 °C for 5 min.

### Production of reconstructed embryos and cloned piglets

Porcine ovaries were obtained from a local slaughterhouse (Nonghyup Moguchon, Gimje, Korea). Cumulus-oocyte complexes (COCs) were collected and washed in Tyrode’s lactate-Hepes containing 0.1 % (w/v) polyvinyl alcohol. Oocytes with several layers of cumulus cells were selected and washed three times in TCM-199 (GIBCO) supplemented with 0.1 % polyvinyl alcohol (w/v), 3.05 mM d-glucose, 0.91 mM sodium pyruvate, 0.57 mM cysteine, 0.5 µg/ml luteinizing hormone, 0.5 µg/ml follicle stimulating hormone, 10 ng/ml epidermal growth factor, 10 % porcine follicular fluid (pFF), 75 µg/ml penicillin G, and 50 µg/ml streptomycin (maturation medium). For in vitro maturation, 50 COCs were transferred into 500 µL of maturation medium in a four-well dish (Nunc, Roskilde, Denmark). The oocytes were matured for 40 h at 38.5 °C under 5 % CO_2_ in air. Somatic cell nuclear transfer was performed as follows (Mezrich et al. 2003; Hauschild et al. 2011). Briefly, matured oocytes in manipulation medium supplemented with 5 µg/ml cytochalasin B were enucleated by aspirating the first polar body, metaphase II chromosomes, and a small amount of surrounding cytoplasm. The freshly-thawed donor cells treated with Roscovitine were inserted into the perivitelline space. The karyoplast-cytoplast complexes were placed into 0.2-mm diameter wire electrodes (1 mm apart) of a fusion chamber covered with 0.3 M mannitol solution containing 0.1 mM MgSO_4_, 1.0 mM CaCl_2_ and 0.5 mM Hepes. For reconstruction, two DC pulses (1-s interval) of 1.5 kV/cm were applied for 30 µs using an Electro-Cell fusion (Fujihira Industry, Tokyo, Japan). Immediately after the confirmation of fusion using a stereoscope, the reconstructed embryos were transferred into both oviducts of the surrogate on the same day or 1 day after the onset of estrus. Pregnancy was diagnosed on day 28 after embryo transfer and was checked regularly every week by ultrasound examination. The cloned piglets with multiple transgenes were delivered by natural parturition or Caesarean section.

### Analysis of protein expression by western blotting

Total umbilical cord tissue extracts were harvested in RIPA buffer and then centrifuged at 12,000×*g* for 10 min at 4 °C. Total protein was quantified using the BCA protein assay reagent, and then proteins were separated by SDS-PAGE and transferred to a PVDF membrane. After blocking in 5 % milk in Tris-buffered saline containing Tween 20, the membrane was incubated with the specific primary antibody including human DAF (1:1000, #GTX103951, GeneTex, Irvine, CA, USA), CD39 (1:1000, #HPA014067, Sigma), TFPI (1:1000, #HPA005575, Sigma), C1-INH (1:1000, #GTX105316, GeneTex), TNFAIP3 (1:1000, #ab92324, Abcam, Cambridge, MA, USA), and β-actin (1:1000, #4967, Cell signaling, Danvers, MA, USA), according to the manufacturer’s recommendation. Signals were detected using ECL reagent according to the manufacturer’s protocol. Densitometry analysis of the blotted membrane was performed using a LAS-3000 lumino image analyzer system (Fujifilm, Tokyo, Japan).

### Necropsy and histopathological analysis

The MGH GT^−(DAF/CD39/TFPI/C1-INH/TNFAIP3)/+^ piglets had normal suckling behaviors and vital signs, and did not show any birth defects after examination by veterinarians. Immediately after the death about 48 h after birth, the MGH transgenic piglets were necropsied to collect tissues and organs such as the brain, spleen, heart, liver, kidney, lung, stomach, testes, thymus, tongue, aorta, and adrenal gland, etc. The same tissues and organs were also collected from the 2-day-old non-transgenic littermates after being humanly euthanized. For the anesthesia and euthanization of control, it was injected with 20 mg/kg of Zoletil^®^ 50 (Virbac, France) and 2.3 mg/kg of Rompun^®^ (Bayer, Toronto, Canada). Tissue samples from control and GT^−(DAF/CD39/TFPI/C1-INH/TNFAIP3)/+^ piglets were fixed in 10 % buffered formalin, embedded in paraffin, and sectioned into 4-µm slices. Sections were stained with hematoxylin and eosin (H&E) and subjected to histopathological analysis. Blood was collected from the jugular vein of GT^−(DAF/CD39/TFPI/C1-INH/TNFAIP3)/+^ and control piglets into tubes containing K2-EDTA (BD Vacutainer^®^). Blood from the cloned littermates, but not targeted, was used as a control. Complete blood counting was performed using a haematology analyser Cell-Dyn^®^ 3700 (Abbott Laboratories, Abbott Park, IL, USA) to determine the level of white blood cells (WBC), including the number of lymphocytes, monocytes, neutrophils, eosinophils, and basophils, as well as, red blood cells (RBC), platelets, haemoglobin, haematocrit, mean corpuscular volume, mean corpuscular haemoglobin concentration, and mean corpuscular haemoglobin.

### Immunohistochemistry

Immunohistochemistry was carried out by the general procedures for paraffin-embedded section on the slides (Lee et al. 2006). For antigen retrieval, slides were placed in citrate buffer and heated in a microwave oven, then washed in dPBS. After treatment 0.3 % Triton X-100 for 10 min, sections were blocked in two steps; firstly, all sections were incubated with peroxidase blocking solution (0.3 % H_2_O_2_ in methanol) to block the endogenous peroxidase for 10 min, and next, they were incubated with serum-free blocking solution (#X0909, Dako, Denmark) for 1 h. The same primary antibodies (dilution 1/100) that have been validated by western blot analysis were incubated overnight. After washing with 0.3 % Tween 20 in dPBS, the sections were incubated with HRP-conjugated anti-rabbit secondary antibody for 1 h (1:200, #sc-2004, Santa Cruz, USA). The human proteins in pig tissues were visualized by using DAB (3,3′-Diamino-benzidine, #K3468, Dako) as a substrate for 5 min. Images of DAB stained tissue sections were captured under a light microscopy. Hematoxylin (#CS700, Dako) was used for counter staining.

### Statistical analysis

For statistical analysis, the GraphPad Prism 5.0 software package (GraphPad Software, La Jolla, CA, USA) was used. Data were analyzed by equal variance, two-tailed Student’s *t* test. *p* values < 0.05 were considered statistically significant.

## Results and discussions

### Establishment of pig cells expressing multiple human transgenes

During the first step to develop a transgenic piglet expressing multiple human genes to minimize graft rejection when transplanted in the primates, we constructed a polycistronic vector to express the following multiple human genes: hDAF, hCD39, hTFPI, hC1-INH, and TNFAIP3, in this order. Based on the findings that most acute vascular rejections are characterized by endothelial cell activation and cellular damage resulting in thrombotic microangiopathy, expression of human transgenes was constructed to be specific in the endothelium of the organ source piglets under the control of an ICAM2 promoter. Moreover, to deplete the α-Gal antigen expression in the pig fibroblast cells, 5′ and 3′ fragments of exon 9 of the GT gene were added to both sides of the vector, respectively, to induce a chromosomal knock-in (KI) of the GT gene by homologous recombination (Fig. 1a). To verify the expression of each transgene by the 2A system, RT-PCR was performed with cDNA prepared from porcine aortic endothelial cells (PAECs) transiently transfected with ICAM2-2A5 as the template and primers specific for each of the open reading frames of human transgenes (Fig. 1b). We established the KI transgenic fibroblast cell lines using the constructed vector. Established transgenic fibroblast cell lines were confirmed by genomic PCR on the junction region between 0.8 kb of promoter and hDAF, and 1 kb of 5′ and 1.1 kb of 3′ exon 9 fragment of the GT gene using specific primer sets, respectively (Fig. 1c). The GT^−(DAF/CD39/TFPI/C1-INH/TNFAIP3)/+^ piglet cell lines were cultured and completely characterized with the aforementioned methods and used in somatic cell nuclear transfer to re-clone the clones.

### Generation of transgenic piglets

**Fig. 2:**
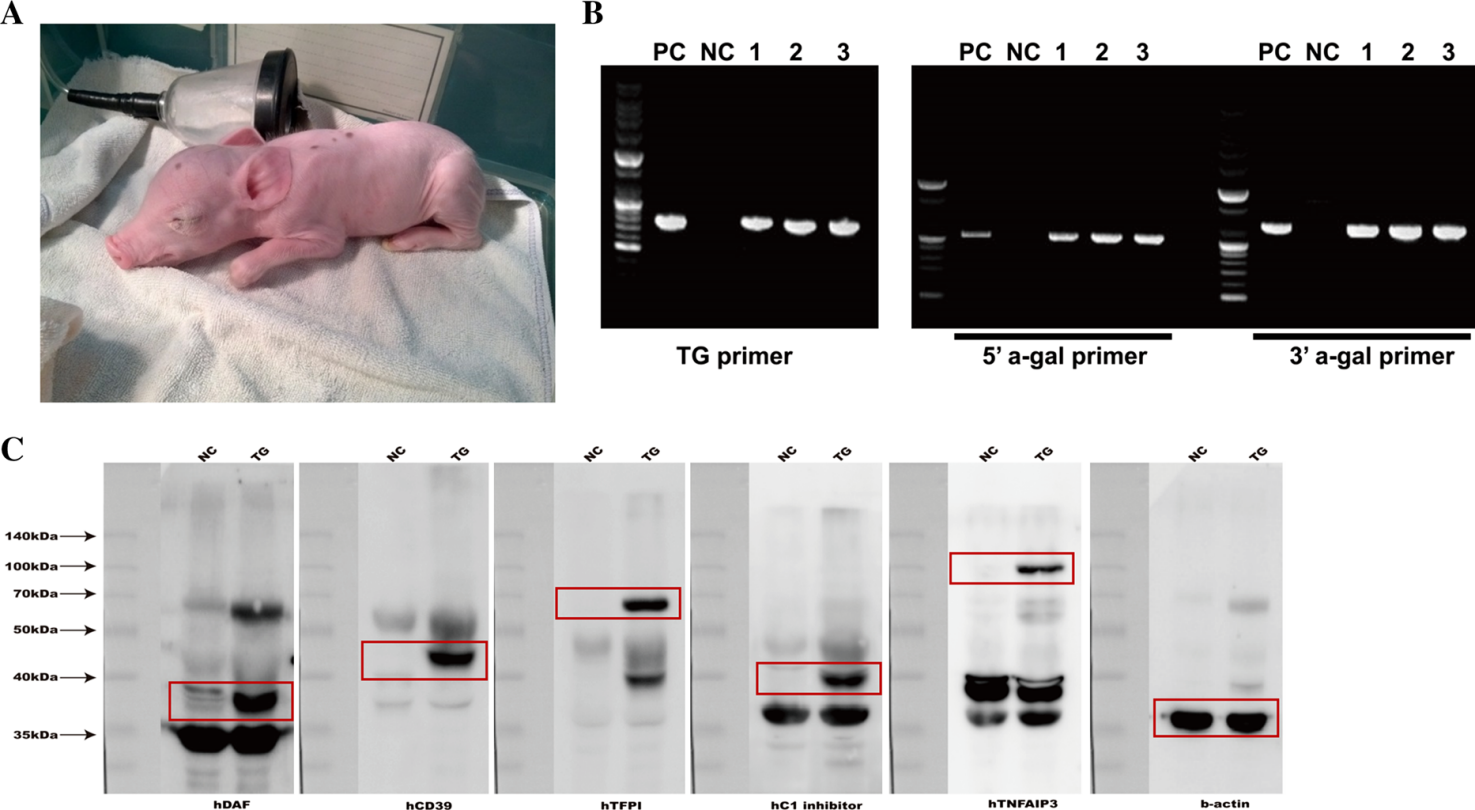
Development of multiple gene expression pigs with GT knock-out. **a** The newborn GT^−(DAF/CD39/TFPI/C1-INH/TNFAIP3)/+^ Massachusetts General Hospital (MGH) piglet delivered by Caesarean section. **b** Genotyping of the piglets by RT-PCR. Three of the multiple transgenic piglets were confirmed genetically with the three primer sets. *PC* positive control which is genomic DNA from cell line #2; *NC* negative control which is normal pig genomic DNA; 1, 2 and 3: genomic DNA from Knock-in pig #1, 2, and 3. **c** western blot analysis. The knock-in piglets expressed the multiple human genes like the constructed vector.* NC* negative control which is a normal pig;* TG* a newborn GT^−(DAF/CD39/TFPI/C1-INH/TNFAIP3)/+^ Massachusetts General Hospital (MGH) piglet. Red boxes represent the exact protein sizes

Individual productivities of cloned piglets were described in Table 1. The pregnancy (7/15 vs. 3/4) and delivery (5/15 vs. 1/4) rates were comparable between cloning and re-cloning donor cells, respectively. Thirteen cloned piglets were born around days 114–117 of pregnancy by Caesarean section or natural parturition (Table 2). Five (cloning + re-cloning) out of 13 cloned piglets were confirmed as the GT^−(DAF/CD39/TFPI/C1-INH/TNFAIP3)/+^ piglets by genomic PCR with the above mentioned methods (Fig. 2a, b). In addition, our data shows some technological improvement in the cloning efficiency using MGH minipigs. The MGH minipig was established for organ transplantation studies across the reproducible homozygous major histocompatibility complex, called the swine leukocyte antigen (Sachs et al. 1976). Although reproductive characteristics of MGH minipigs are lower than those of domestic pigs (Conley et al. 1988; Mezrich et al. 2003) due to the inheritance of homozygous major histocompatibility complex in the highly inbred MGH minipigs, recipient-based cloning efficiency (no. of newborn piglets/transferred cloned embryos) of GT^−(DAF/CD39/TFPI/C1-INH/TNFAIP3)/+^ cloned MGH piglets was highly increased to 5.33 % in the present study when compared to the value of previous reports: 2.54 % in GT^–MCP/+^ (Hwang et al. 2013) and 0.2 % in GT^−/+^ cloned MGH minipigs (Hauschild et al. 2011).

**Table 1:** Pregnancy and delivery rate by donor cell types

Types	No. of embryos transferred	No. of surrogates		
		Transferred	Pregnant^a^	Delivered^b^
Cloning	122 ± 29	15	7	5
RE-cloning	123 ± 29	4	3	1

Data were expressed as mean ± standard deviation. Cloning: primary TG cell; RE-cloning: TG cells originated from cloning pig

^a^Pregnancy was confirmed by gestation sac formation using ultrasound examination at 28 days after embryo transfer

^b^No. of surrogates delivered/No. of surrogates pregnant

**Table 2:** Full-term development of multi-transgenic cloned piglets

Types	No. of surrogates	Delivery status^a^	No. of piglets born			Targeting
			Total	Survival	Dead^b^	
Cloning	1	117 (C-sec)	4	2	2	1
	2	117 (C-sec)	1	1		–
	3	116 (natural)	5	5		2
	4	117 (C-sec)	1		1	–
	5	114 (C-sec)	1	1		1
Re-cloning	6	114 (C-sec)	1	1		1

Cloning: primary TG cell; Re-cloning: TG cells originated from cloning pig

All five targeted piglets died about 48 h after birth without specific symptoms

^a^Pregnancy periods after transfer of multi-transgenic cloned embryos

^b^Piglets that died within 1 day

### Histopathological and hematological investigation

Behavior and milk consumption of the MGH GT^−(DAF/CD39/TFPI/C1-INH/TNFAIP3)/+^ piglets were similar to previously generated piglets (Ahn et al. 2011; Kwon et al. 2011; Hwang et al. 2013). Gross observation at necropsy shows no significant pathological findings compared to non-transgenic littermates. Histological examination of organs, including the brain, spleen, heart, liver, kidney, lung, stomach, testes, thymus, tongue, aorta, and adrenal gland, did not show different pathological changes (data not shown). However, a complete blood count test of the MGH transgenic piglets showed significantly lower blood cell counts for WBCs (*p* = 0.001), including neutrophils (*p* = 0.035), lymphocytes (*p* = 0.008), eosinophils (*p* = 0.019), and basophils (*p* = 0.005), while no significant changes were observed in monocyte counts than those of non-transgenic littermates. Platelet counts (*p* = 0.008) were also significantly decreased in the transgenic piglets. On the other hand, RBC counts, hemoglobin, hematocrit, and mean corpuscular volume were not significantly changed, although a significant decrease in mean corpuscular hemoglobin (*p* = 0.017) was observed in the transgenic piglets (Table 3). These data suggest that transgenic expression of those human genes in pigs impairs hematopoiesis, but does not or minimally affects erythropoiesis.

**Table 3:** Mean values of hematological parameters in the newborn cloned piglets

Abbreviation	Units	Reference^a^	Multi-TG (n = 2)	Control^b^ (n = 2)
WBC (*p* = 0.001)	×10^3^/µl	16.8–32.4	1.3 ± 0.66	13.6 ± 0.35
Neutrophils (*p* = 0.035)	×10^3^/µl	7.6–24.6	0.14 ± 0.01	3.77 ± 1.44
Lymphocytes (*p* = 0.008)	×10^3^/µl	3.42–12.7	0.53 ± 0.41	7.65 ± 1.21
Monocytes	×10^3^/µl	0.2–3.2	0.58 ± 0.25	1.27 ± 0.34
Eosinophils (*p* = 0.019)	×10^3^/µl	0.0–0.7	0.03 ± 0	0.67 ± 0.18
Basophils (*p* = 0.005)	×10^3^/µl	0.0–0.5	0.03 ± 0.004	0.16 ± 0.02
Platelet (*p* = 0.008)	×10^3^/µl	200–800	74.9 ± 27.9	232 ± 2.8
RBC	×10^3^/µl	6.4–9.3	5.98 ± 2.59	7.38 ± 1.1
Hemoglobin	g/dl	11.4–13.5	10.19 ± 4.4	13.7 ± 2.47
Hematocrit	%	35–41	34.95 ± 15.5	40.3 ± 6.2
Mean corpuscular volume	fl	48.1–63.9	58.35 ± 0.64	54.7 ± 0.35
Mean corpuscular hemoglobin	pg	13.7–18.8	17.0 ± 0	18.5 ± 0.7
Mean corpuscular hemoglobin concentration	%/g/dl	30.8–36	29.2 ± 0.28	33.9 ± 0.84

Two-tailed Student’s *t* test (*p* < 0.05)

^a^Laboratory Animal Medicine 2nd edition, Edited by: Fox et al., Chapter 15—Biology and Diseases of Swine

^b^Control: non-transgenic cloned littermates

Targeted transgenic protein expression was confirmed by western blot analysis using the umbilical cord of the GT^−(DAF/CD39/TFPI/C1-INH/TNFAIP3)/+^ piglets (Fig. 2c). In addition, the protein expressions were analyzed by immunohistochemical analysis. The hCD39, hTFPI, and hTNFAIP3 proteins were obviously detected in kidney, liver and aorta of the piglets, but not in those of the control wild-type piglet (Fig. S1). However, unlike western blot analysis, some of the tansgenic proteins, such as hDAF and hC1-INH, were not or weakly detected in those tissues, possibly due to low affinities of the antibodies on the tissue sections. Because all five human proteins were designed to be expressed as a single gene product and subsequent western blot analysis of piglet tissues has confirmed that individual proteins are successfully produced by precise cleavage at the ‘self-cleaving’ 2A peptide sequences, hDAF and hC1-INH proteins are certainly expressed in the transgenic piglets. This approach is practically advantageous in the transgenic expression of more than one protein from a single vector than the conventional approach, which requires the use of internal ribosome entry sequences or additional promoters, often leading to the differential expression level of proteins.

There have been previous reports demonstrating that transgenic expression of human DAF, CD39, TFPI, or TNFAI3 genes except C1-INH, alone or in combination, in pigs show no significant pathology in blood cells, such as leucopenia and thrombocytopenia. Moreover, the expression of C1-INH protein appears not to be directly correlated with leucopenia and thrombocytopenia because it plays a protective role against complement-mediated activation and subsequent cellular damages in endothelial cells (Dalmasso and Platt 1993; Fiane et al. 1999). In the current studies, the ectopic expression of all five transgenes in piglets, however, resulted in leucopenia and thrombocytopenia. Because blood leukocytes are the first line of defense against infection, and platelets play crucial roles to stop bleeding after blood vessel injuries, leucopenia and thrombocytopenia may be a major cause of the death of the transgenic piglets.

Among five transgenes, TFPI has been reported to promote platelet aggregation, leading to a remarkable decrease in platelet count (Nishioka et al. 2002; Ellery et al. 2015), which could partially explain the low platelet count in our transgenic piglets. Additional contributors to the decreased platelets in the pigs might be CD39 and DAF overexpression since transgenic production of human CD39 and DAF in mice resulted in slow clotting of human blood (Dwyer et al. 2006; Miwa et al. 2010).

Among the five target proteins that are consistently expressed in endothelial cells of the transgenic piglets, secretory proteins (TFP1 and C1-INH) that are circulating in the blood may systemically affect development of immune cells. Another possible explanation could be based on the finding that the human ICM2 promoter drove expression of transgene in endothelial cells and leukocytes, including neutrophils and monocytes (Cowan et al. 1996, 2003). Similar to the human promoter activities in the aforementioned transgenic mice, it is possible that the porcine ICAM2 promoter might be active in endothelial cells and leukocytes, but not in erythrocytes, consequently driving production of the five target proteins in both endothelial cells and leukocytes and affecting hematopoiesis in the transgenic piglets.

Very recently, an abstract was reported that the survival of renal xenograft from a donor transgenic pig with 6 transgenes (GT KO/CD46/DAF/EPCR/TBM/CD39) to baboon survived for 135 days (Ayares et al. 2015). The survival period of the 6-gene TG pig was shorter than the GT KO/CD55 transgenic pig (>227 days). It is difficult to compare the survival results only in the survival period because of many different circumstances in both groups. Kidneys from GT KO/CD55/CD59/HTF pigs were transplanted into four baboons, and all of them were rejected within 10 days (Hawthorne et al. 2015).

Although organs from GT KO pigs with transgenic expression of multiple human genes involved in the immune and coagulation systems has resulted in an extended xenograft survival in primates (Le Bas-Bernardet et al. 2011; Ayares et al. 2015; Hawthorne et al. 2015), much remains to be improved in generating multiple transgene expression in pigs for xenograft survivals, as the survival of xenografts is highly dependent upon many factors, including the kinds of transgenic genes, expression levels, and recipient and organ types.

Our current studies confront health issues of donor pigs with genetic modification of multiple genes that could bring beneficial aspects associated with immune suppression and anti-clotting of blood. Further studies are needed in both appropriate genetic modifications and immune responses in order to further extend survival.

## Conclusions

In the present communication, we produced the transgenic piglets capable of simultaneously expressing five different human genes without α-Gal antigen expression [GT^−(DAF/CD39/TFPI/C1-INH/TNFAIP3)/+^]. Accordingly, full understanding of the molecular mechanism involved in xenograft rejection, as well as the development of efficient technology to produce genetically manipulated organ-source piglets that harbor multiple human genes, are essentially required to improve the success rates of xenotransplantation in the future. However, several issues still remain to be resolved for clinical application of these strategies, including the selection of transgenes and potential pathological issues, such as leucopenia and thrombocytopenia, presented in this study. Better understanding of the biology and functional roles of candidate transgenes may help to develop safe and efficient approaches for the genetic manipulation of multi-TG pigs and for the successful xenotransplantation in the future.

## Electronic supplementary material

Below is the link to the electronic supplementary material.

Supplementary material 1 (PPT 2835 kb)

Supplementary material 2 (PDF 56 kb)

## References

[CR1] Ahn KS, Kim YJ, Kim M (2011). Resurrection of an alpha-1,3-galactosyltransferase gene-targeted miniature pig by recloning using postmortem ear skin fibroblasts. Theriogenology.

[CR2] Ayares D, Vaught T, Ball S et al (2015) Six-gene multi-transgenic pigs serve as source animals to address innate and chronic rejection, inflammation, and coagulopathy in organ xenografts. In: Transplantation, pp s59–s59

[CR3] Conley AJ, Jung YC, Schwartz NK (1988). Influence of SLA haplotype on ovulation rate and litter size in miniature pigs. J Reprod Fertil.

[CR4] Cowan PJ, Shinkel TA, Witort EJ (1996). Targeting gene expression to endothelial cells in transgenic mice using the human intercellular ahdesion molecule 2 promoter. Transplantation.

[CR5] Cowan PJ, Shinkel TA, Fisicaro N (2003). Targeting gene expression to endothelium in transgenic animals: a comparison of the human ICAM-2, PECAM-1 and endoglin promoters. Xenotransplantation.

[CR6] Dalmasso AP, Platt JL (1993). Prevention of complement-mediated activation of xenogeneic endothelial cells in an in vitro model of xenograft hyperacute rejection by C1 inhibitor. Transplantation.

[CR7] Daniel S, Arvelo MB, Patel VI (2004). A20 protects endothelial cells from TNF-, Fas-, and NK-mediated cell death by inhibiting caspase 8 activation. Blood.

[CR9] Dwyer KM, Mysore TB, Crikis S (2006). The transgenic expression of human CD39 on murine islets inhibits clotting of human blood. Transplantation.

[CR10] Ellery PER, Maroney SA, Cooley BC (2015). A balance between TFPI and thrombin-mediated platelet activation is required for murine embryonic development. Blood.

[CR11] Fiane AE, Videm V, Johansen HT (1999). C1-inhibitor attenuates hyperacute rejection and inhibits complement, leukocyte and platelet activation in an ex vivo pig-to-human perfusion model. Immunopharmacology.

[CR12] Griesemer AD, Hirakata A, Shimizu A (2009). Results of gal-knockout porcine thymokidney xenografts. Am J Transplant.

[CR13] Hauschild J, Petersen B, Santiago Y (2011). Efficient generation of a biallelic knockout in pigs using zinc-finger nucleases. Proc Natl Acad Sci USA.

[CR14] Hawthorne W, Salvaris E, Hawkes J et al (2015) Enhanced survival of GalT KO/hCD55-hCD59 pig renal xenografts in baboons. In: Transplantation, pp S237–S237

[CR15] Higginbotham L, Mathews D, Stephenson A et al (2015) Long-term survival of pig-to-primate renal xenotransplant using costimulation-blockade immunosuppression. In: Transplantation, pp s72–s73

[CR16] Hwang S, Oh KB, Kwon D-J (2013). Improvement of cloning efficiency in minipigs using post-thawed donor cells treated with roscovitine. Mol Biotechnol.

[CR18] Kwon DJ, Kwak TU, Oh KB et al (2011) Effects of donor cell treatments on the production of transgenic cloned piglets. Reprod Dev Biol

[CR19] Lai L (2002). Production of alpha-1,3-galactosyltransferase knockout pigs by nuclear transfer cloning. Science.

[CR20] Lambrigts D, Sachs DH, Cooper DK (1998). Discordant organ xenotransplantation in primates: world experience and current status. Transplantation.

[CR21] Le Bas-Bernardet S, Tillou X, Poirier N (2011). Xenotransplantation of galactosyl-transferase knockout, CD55, CD59, CD39, and fucosyl-transferase transgenic pig kidneys into baboons. Transplant Proc.

[CR22] Lee JW, Beebe K, Nangle La (2006). Editing-defective tRNA synthetase causes protein misfolding and neurodegeneration. Nature.

[CR23] Lee HJ, Lee BC, Kim YH (2011). Characterization of transgenic pigs that express human decay accelerating factor and cell membrane-tethered human tissue factor pathway inhibitor. Reprod Domest Anim.

[CR24] McGregor CG, Teotia SS, Byrne GW (2004). Cardiac xenotransplantation: progress toward the clinic. Transplantation.

[CR25] Mezrich JD, Haller GW, Arn JS (2003). Histocompatible miniature swine: an inbred large-animal model. Transplantation.

[CR26] Miwa Y, Yamamoto K, Onishi A (2010). Potential value of human thrombomodulin and DAF expression for coagulation control in pig-to-human xenotransplantation. Xenotransplantation.

[CR27] Mohiuddin M, Singh A, Corcoran P et al (2015) Critical need of continuous co-stimulation blockade with anti CD40 antibody (2C10. R4) for long-term maintenance of GTKO. HCD46. hTBM pig cardiac xenograft survival in baboons. In: Transplantation, pp S236–S236

[CR28] Nishioka T, Yokota M, Hino M (2002). Tissue factor pathway inhibitor can interact with platelets. J Immunol Methods.

[CR29] Oropeza M, Petersen B, Carnwath JW (2009). Transgenic expression of the human A20 gene in cloned pigs provides protection against apoptotic and inflammatory stimuli. Xenotransplantation.

[CR30] Sachs DH, Leight G, Cone J (1976). Transplantation in miniature swine: i. Fixation of the major histocompatibility complex. Transplantation.

[CR31] Wheeler DG, Joseph ME, Mahamud SD (2012). Transgenic swine: expression of human CD39 protects against myocardial injury. J Mol Cell Cardiol.

[CR32] Yamada K, Yazawa K, Shimizu A (2005). Marked prolongation of porcine renal xenograft survival in baboons through the use of alpha1,3-galactosyltransferase gene-knockout donors and the cotransplantation of vascularized thymic tissue. Nat Med.

