# Supplementary material for: Generation of α-1,3-galactosyltransferase knocked-out transgenic cloned pigs with knocked-in five human genes
Source: Transgenic Res. 2016 Aug 23;26(1):153–63. doi: 10.1007/s11248-016-9979-8 (PMC5243873; doi:10.1007/s11248-016-9979-8)
Supplement: Supplementary file 1 — Supplementary material 1 (PPT 2835 kb) [file 11248_2016_9979_MOESM1_ESM.ppt]

## Slide 1
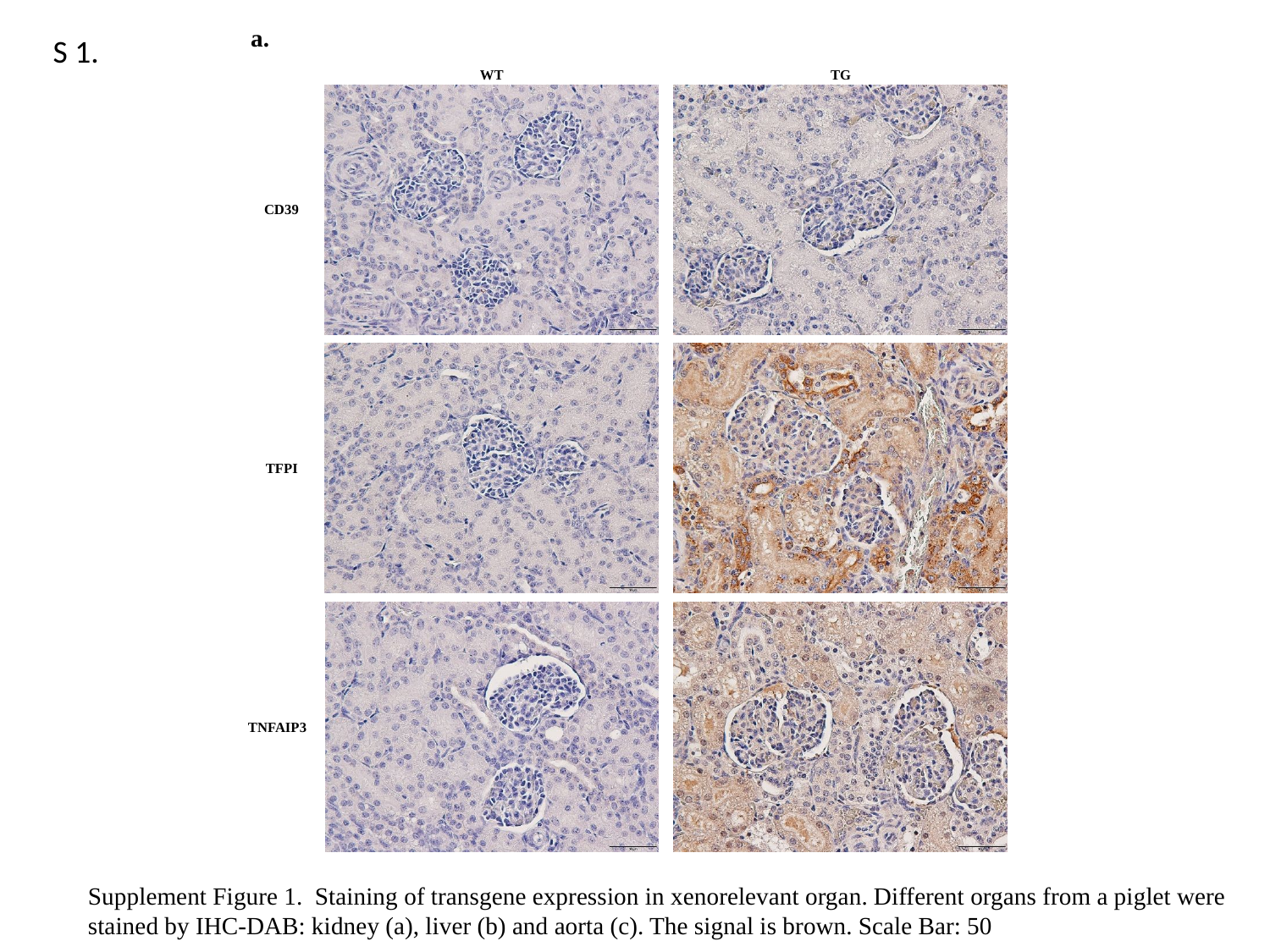

a.
S 1.
WT
TG
CD39
TFPI
TNFAIP3
Supplement Figure 1. Staining of transgene expression in xenorelevant organ. Different organs from a piglet were stained by IHC-DAB: kidney (a), liver (b) and aorta (c). The signal is brown. Scale Bar: 50㎛

## Slide 2
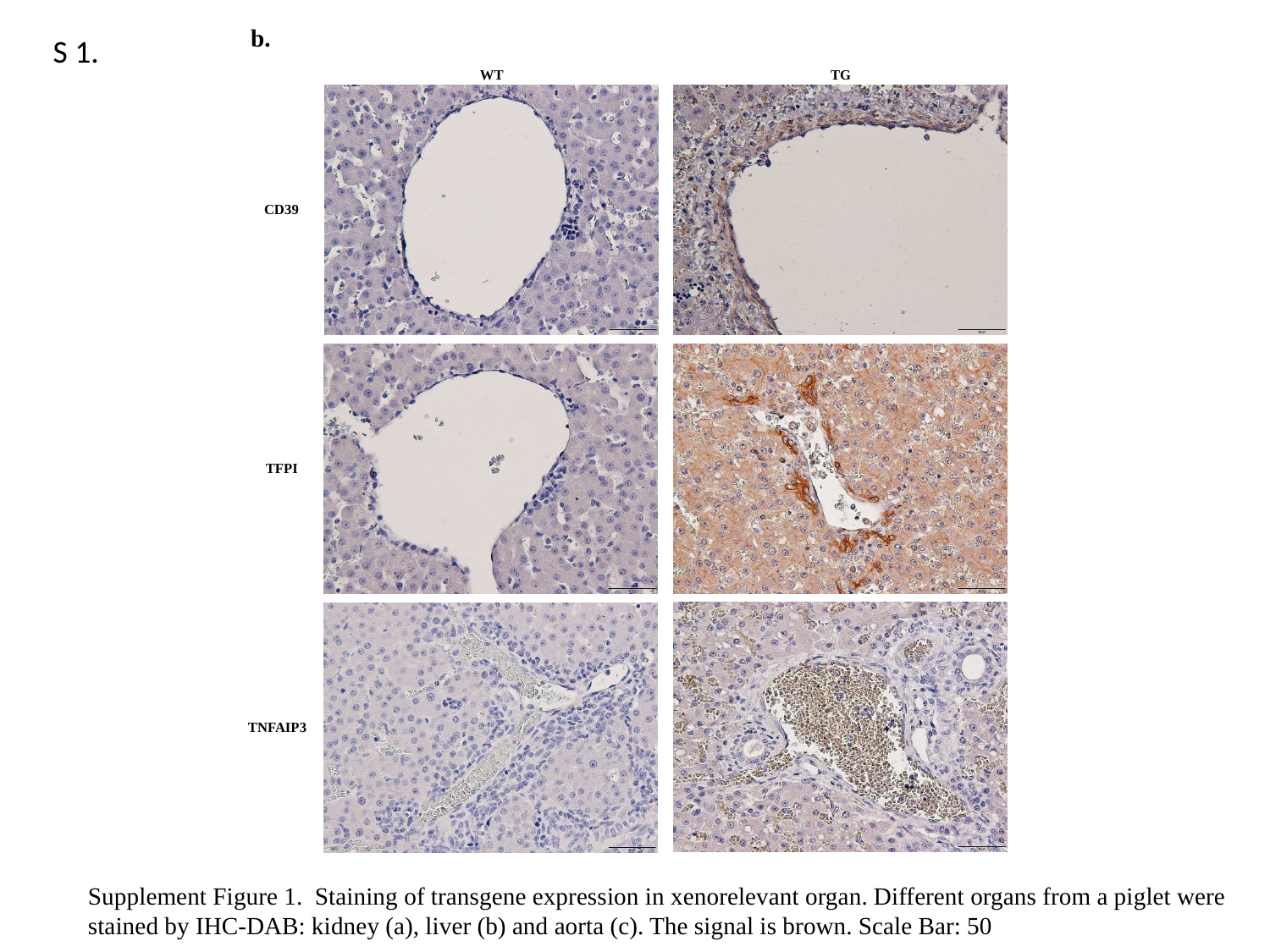

b.
S 1.
WT
TG
CD39
TFPI
TNFAIP3
Supplement Figure 1. Staining of transgene expression in xenorelevant organ. Different organs from a piglet were stained by IHC-DAB: kidney (a), liver (b) and aorta (c). The signal is brown. Scale Bar: 50㎛

## Slide 3
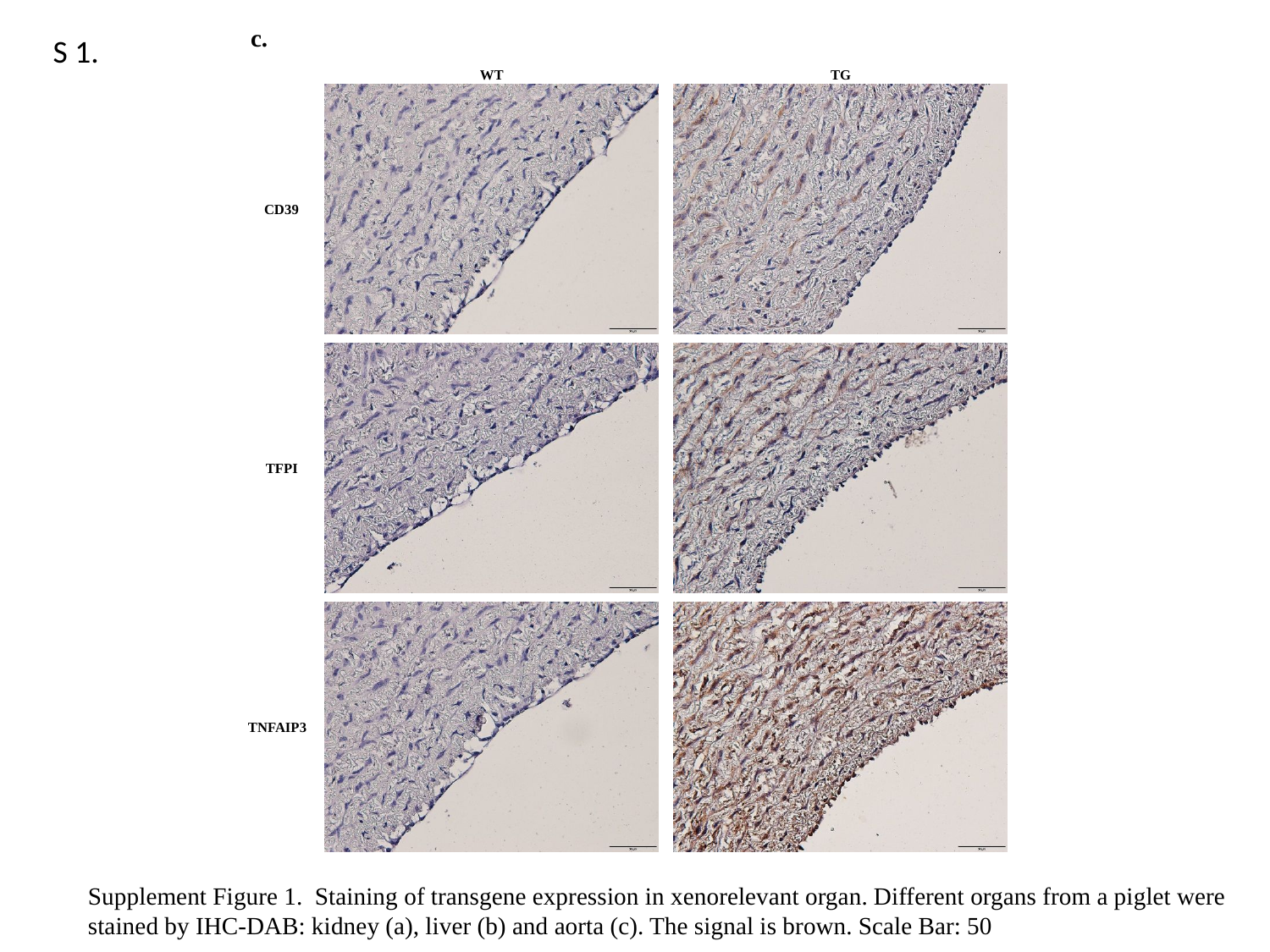

c.
S 1.
WT
TG
CD39
TFPI
TNFAIP3
Supplement Figure 1. Staining of transgene expression in xenorelevant organ. Different organs from a piglet were stained by IHC-DAB: kidney (a), liver (b) and aorta (c). The signal is brown. Scale Bar: 50㎛
