# Supplementary material for: Generation of α-1,3-galactosyltransferase knocked-out transgenic cloned pigs with knocked-in five human genes
Source: Transgenic Res. 2016 Aug 23;26(1):153–63. doi: 10.1007/s11248-016-9979-8 (PMC5243873; doi:10.1007/s11248-016-9979-8)
Supplement: Supplementary file 2 — Supplementary material 2 (PDF 56 kb) [file 11248_2016_9979_MOESM2_ESM.pdf]

Table 1. PCR primers

Regions	Sense primer	Antisense primer
ICAM2 promoter	ctcgagttcggagactggatgtatcatgat	gtcgaccttgagtggcacccacgggct ggcaggaaa
hCD55	agatatcgcgcggccgagcgtgcccgcgg	acctaggtctcttagcacgagtcagcaa gcccatggttac
hCD39	atacgtaacaaaggagtctaacgtgaaga	aactagttctcttagctcgtaccatatc tttccagaaatat
hTFPI	acccgggacaatgaagaaagtacatgcac	acctaggtctcttagctcgacataggca tgaaatgctatcc
hC1 inhibitor	aaggcctgcctccaggctgaccctgctg	agctagctctcttagctcgggccctggg gtcatatactcg
hTNFAIP3	atcgcgacaagtccttcctcaggctttg	agtcgacttagccatacatctgcttgaa
5' α-Gal	gcggccgcggtgttgctgtggctgtggca	ctcgagatccaccatgatgtaaaagatg
3' α-Gal	gtcgaccaacaaacccactaaaat ctta	ggcgcgccctccatgccctgtgtctcttag

Table 2. PCR primers for confirmation of cell line

Regions	Sense primer	Antisense primer	Tm(°C)
TG	gagcatgccaggatgacaaggtta	tgccacctggtacatcaatctgac	55
5' KI	tgctcagtgggttaaaggatcaag	catccctggaaacagtgcgcaggt	65
3' KI	attgagatgcatgctttgcatact	ttctaagacagtgctgctctagtt	50
Pig GAPDH	cctccactacatggtctacat	tcatgagtccctccacgatg	57
hCD55	agatgtacctaatgcccagcc	ccacctggtacatcaatctga	58
hCD39	ccaggatcattactggccaag	aggtcacttacgttcactacc	58
hTFPI	cggagttgccaccactgaaac	ttccataattatccacctgga	55
hC1 inhibitor	gcaacaacagtgacgccaact	tgggctggaacttggacatct	59
hTNFAIP3	ggaccatggcacaactcatct	ttcatggcagtggtctcactg	58
